# Plant-level carbon accounting of China's pulp and paper industry via multimodal fusion

**DOI:** 10.1016/j.ese.2026.100682

**Published:** 2026-03-06

**Authors:** Song Hu, Huaqing Qi, Zifei Wang, Xiaoyu Wu, Yulin Han, Yi Man

**Affiliations:** a State Key Laboratory of Advanced Papermaking and Paper-Based Materials, South China University of Technology, Guangzhou, 510640, China; b School of Chemistry and Chemical Engineering, South China University of Technology, Guangzhou, 510640, China

**Keywords:** Multimodal, Pulp and paper industry, Plant-scale, Carbon accounting, Carbon reduction potential

## Abstract

Plant-scale industrial carbon accounting is critical for developing targeted emission-reduction policies. However, most assessments of carbon-intensive sectors rely on aggregate statistics, which obscure significant heterogeneity among individual plants. China's pulp and paper industry (PPI), the largest globally, encompasses diverse production processes, raw material inputs, and emission sources. Existing accounting frameworks rely on statistical data and average emission factors within poorly defined system boundaries, which prevents differentiation at the individual plant level. Here, we propose a multimodal data fusion framework that integrates high-resolution remote-sensing imagery with plant textual data to capture structural and operational characteristics undetectable by any single data modality. Applied to 720 pulping and papermaking plants across China, the framework achieves R^2^ values of up to 0.96 across five plant types and estimates total sectoral carbon emissions at 163.6 million tonnes of CO_2_ in 2022, with pronounced regional disparities concentrated in eastern coastal provinces. Analysis of functional-zone contributions further reveals that wastewater treatment areas are a consistent cross-category emission driver, and that just 5% of high-emission plants account for approximately 43% of sectoral emissions—a skewed structure that demands differentiated regulatory intervention. Incorporating regional solar radiation data, rooftop photovoltaic deployment is projected to reduce annual PPI emissions by up to 10.3%, with primary-fiber pulp plants offering the greatest mitigation leverage. Beyond China's PPI, this scalable, data-driven approach provides a transferable blueprint for granular, plant-level carbon accounting in other heterogeneous heavy industries.

## Highlights

• China's 720 pulp and papermaking plants emitted ∼163.6 Mt CO_2_ in 2022, with >60% from eastern coastal provinces.
• A multimodal DeepLabv3+/BERT fusion framework predicts carbon emissions with R^2^ values reaching 0.96.
• Just 5% of high-emission plants account for ∼43% of total sectoral emissions, revealing extreme emission skewness.
• Rooftop photovoltaic deployment could cut annual PPI emissions by up to 16.9 Mt CO_2_ (10.3% of the total).

## Graphical abstract

**Figure:**
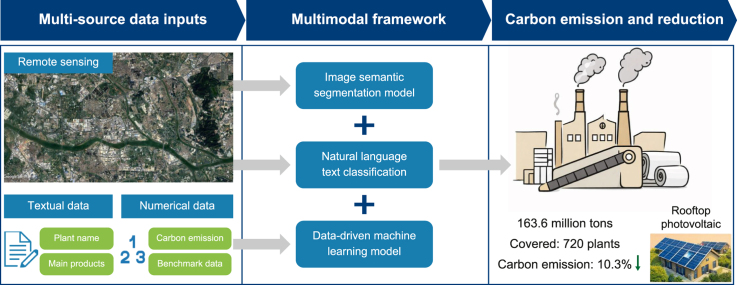

## 1 Introduction

As the world’s largest producer of paper products, China has included the pulp and paper industry (PPI) among the eight key emission-intensive sectors of the national carbon market. The PPI's energy conservation and emissions-reduction process is vital to supporting China’s transition toward sustainable development [1], and carbon accounting for the PPI is the foundation for achieving carbon emissions reduction (CER) [2]. Accurate quantification of carbon emissions and source contributions is thus fundamental for formulating mitigation strategies and policy frameworks.

Previous researchers have assessed PPI carbon emissions at global [3], national [4], and regional [5] scales, thereby informing the development of macro-level policies to mitigate emissions. As fundamental units within the PPI that generate carbon emissions, pulping and papermaking plants (PPPs) represent the most granular scale at which CER policies can be effectively implemented [6]. Achieving sector-wide carbon neutrality in the PPI depends on the decarbonization performance of individual plants [7]. Therefore, there is a need to establish a high-resolution, operationally feasible carbon accounting framework at the plant scale. Such a framework would facilitate the precise identification of emission source distributions and provide a robust scientific basis for the design of differentiated and targeted mitigation strategies.

Carbon emissions from PPPs are commonly estimated by combining energy consumption data with corresponding emission factors. Existing emissions inventories span scales ranging from individual plants [8] to industrial clusters of up to 23 plants [9]. However, energy-based accounting approaches neglect direct carbon emissions from pulping and wastewater treatment processes, resulting in the omission of approximately 20% of total emissions in energy-based emission estimates [10] and potentially introducing substantial biases into total emissions estimations.

In addition to methods based on energy consumption, plant-level carbon emissions can be estimated by integrating publicly available production-capacity data with product-specific carbon-emission factors. This method has been applied to construct large-scale, plant-level carbon emission inventories for the refining [6], steel [11], and primary aluminum smelting [12] industries. It has also been extended to the PPI through life cycle assessment (LCA), which is used to estimate carbon emission factors for major paper products in China [13]. In 2015, by integrating these factors with plant-level production-capacity data, researchers developed a comprehensive carbon-emission inventory covering 814 PPPs across China [14]. In this inventory, production capacity is treated as an indirect indicator of emissions and used to estimate plant-level carbon emissions using product-specific carbon-intensity parameters. It has certain advantages, such as broad applicability and ease of data collection [15]; however, variations in process technology, raw material compositions, and scale effects across plants lead to pronounced differences in product-level carbon emission intensities [16]. These differences undermine the accuracy of carbon emission estimates, even within the same industrial sector. Moreover, this approach is further complicated by inconsistent system boundaries and ambiguous emission attribution to individual plants [17].

Remote-sensing imagery—a high-resolution observational tool—allows the spatial layouts of PPPs, including production buildings, raw material storage zones, and wastewater treatment areas [18], to be identified. Such spatial information supports differentiation among raw material structures and scale-related characteristics across plants, providing spatial data for carbon accounting [19]. However, such imagery, when used alone, often cannot distinguish among different types of PPPs, because plants with similar spatial layouts may differ substantially in product types and emission characteristics. In contrast, textual information captures knowledge that cannot be directly obtained from images, including plant product names and structures. Multimodal data fusion refers to the integration of heterogeneous data from multiple sources or modalities to provide a more comprehensive and accurate representation of a system or process. Multimodal data fusion approaches have been applied to address the limitations of single-source remote-sensing imagery, including urban waste pile detection [20], urban village detection [21], and urban area functional identification [22]. Therefore, building on these advances, we integrated plant-level remote-sensing imagery with product semantic information and used a multimodal fusion framework to improve the accuracy of PPP classification and plant-level carbon emission estimation.

Estimating carbon emissions at the plant level improves the compilation of carbon emission inventories and provides a necessary foundation for differentiated CER management. At the plant scale, currently implementable decarbonization options for PPPs include improving energy efficiency, adjusting the energy mix, increasing waste paper utilization, and deploying carbon capture and storage (CCS) technologies [23].

Optimizing production processes, improving equipment retrofitting, and enhancing thermal and electrical energy efficiency can reduce carbon emissions by 6.8–192.1 kg of carbon dioxide (CO_2_) per ton of paper [24]. However, substantial heterogeneity in production equipment lifetimes and process configurations leads to pronounced differences in energy consumption patterns across PPPs. Consequently, establishing a unified retrofitting strategy that does not disrupt normal production is challenging [25]. Substituting recycled fibers for virgin wood pulp can reduce carbon emissions by approximately 25% [26], but China’s ban on imported waste paper has greatly reduced recycled fiber resources, resulting in an estimated supply gap of around 30 million tons [13]. CCS technology can mitigate the high-concentration direct emissions from thermal power units in PPPs. However, in practice, most Chinese PPPs do not use thermal power units, and their emissions mainly arise indirectly from electricity consumption and steam supply [27]. Furthermore, the absence of centralized emissions sources, together with high capital investment requirements, elevated operational costs, and insufficient CO_2_ transport and storage infrastructure, severely restricts the application of CCS at the plant level [28].

At present, optimizing the energy structure at the plant level seems to be the most feasible pathway for reducing carbon emissions in China’s PPI [29]. Increased use of renewable energy, such as from green electricity and photovoltaic (PV) systems, can reduce the indirect emissions associated with electricity consumption [30]. However, the emissions reduction benefits of green electricity depend on the cleanliness of the regional power grid and the external energy structure [31]. In regions dominated by fossil fuel power generation, the mitigation effect of green electricity substitution is limited. In contrast, PV power generation offers great potential and flexibility for industrial decarbonization. Previous studies have demonstrated that PV systems installed on plant rooftops or within industrial parks can reduce a portion of purchased electricity consumption [32,33]. The roofs of PPPs are typically spatially distributed, underutilized, and centrally managed, making them particularly suitable for deploying distributed PV systems. When combined with energy storage systems, PV systems facilitate tiered energy utilization, thereby accelerating the PPI decarbonization process [34]. Recent studies on PPI decarbonization strategies based on PV power have primarily focused on national-scale assessments [35]. Few systematic assessments of the potential of PV power generation for plant-level decarbonization have been conducted.

To address unclear boundaries and substantial estimation biases in existing carbon accounting methods for PPPs, we developed a multimodal carbon accounting framework by integrating information from remote-sensing images and textual data. First, we developed a multimodal classification strategy based on the DeepLabv3+ semantic segmentation model and bidirectional encoder representations from the transformers (BERT) text-based model. This facilitated the accurate identification and categorization of PPPs based on their spatial and semantic features. Second, we developed a data-driven, differentiated carbon-accounting model to estimate 2022 carbon emissions for 720 PPPs and to provide a plant-level emissions inventory for China’s PPI. Finally, we incorporated solar radiation and geographic data to assess the potential of deploying PV systems within existing PPPs to reduce carbon emissions and support actionable decarbonization solutions at the plant level.

## 2 Methodology

**Fig. 1:**
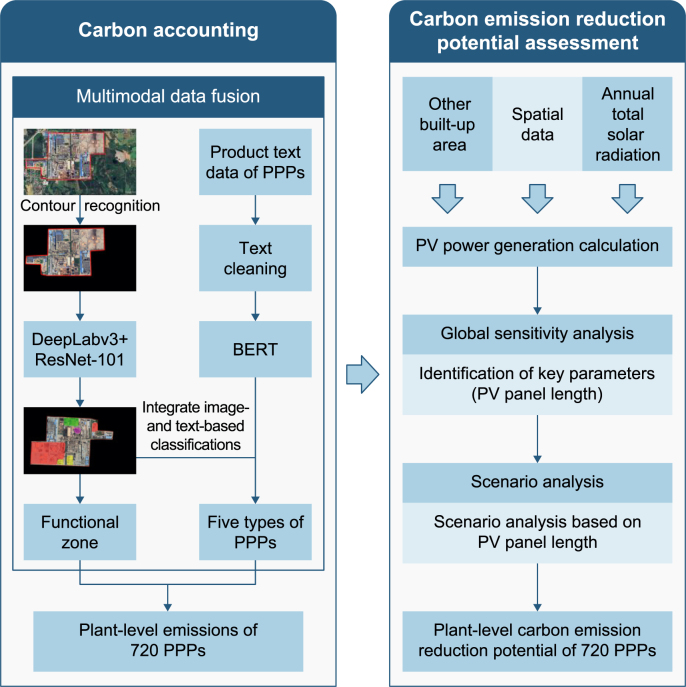
Framework of this study. The left panel shows the carbon accounting process. Image- and text-based classification results are integrated to determine functional zones and categorize pulping and papermaking plants (PPPs) into five types, enabling the estimation of plant-level carbon emissions for 720 PPPs. The right-hand panel illustrates the assessment of the potential to reduce carbon emissions, where the generation of photovoltaic (PV) power is estimated, and global sensitivity and scenario analyses are conducted to evaluate the potential to reduce carbon emissions at the plant level under different panel lengths. BERT: bidirectional encoder representations from the transformers.

This study aimed to address key carbon-accounting issues in China’s PPI, including low spatial resolution of region-level statistical data, limited data dimensions, and challenges in accounting for plant heterogeneity. By integrating image recognition, natural language processing, and data-driven modeling, we developed a framework for estimating plant-level carbon emissions. The overall framework of this study consists of two core components—carbon accounting and an assessment of CER potential (Fig. 1). These two components are described in detail below:

***Carbon accounting.*** First, we delineated the boundaries of each PPP and applied contour extraction techniques to identify the regions of interest (ROIs) corresponding to plant areas from remote-sensing imagery. We then used a semantic segmentation model based on DeepLabv3+ to segment and identify functional zones within each plant, enabling quantification of the area associated with each functional region. Simultaneously, we cleaned the textual data to remove noise and analyzed it using a BERT model to generate plant-classification results. We subsequently integrated the outputs of the image-based and text-based analyses at the decision level. Specifically, we used image-based information mainly to distinguish between primary fiber pulp plants and recovered fiber pulp plants, whereas we used textual data to classify other types of PPPs. Based on the final plant categories, we developed an area-based carbon accounting model for each PPP, using functional-zone measurements and corresponding carbon-emission data.

***CER potential assessment.*** Based on the PPP area, geographical location, and solar radiation data, we developed a mechanistic model to evaluate the CER potential of rooftop PV power generation. We then employed the Kucherenko index to conduct a global sensitivity analysis (GSA) and quantify the influence of the model input variables and their interactions on the outputs. The Kucherenko index uses a quasi-random Sobol sequence with high spatial sampling efficiency to ensure that the control variables are mutually independent. This not only allows the first-order effects of individual factors to be assessed but also captures interactions between variables. When the sensitivity index exceeds 0.1, the factor is considered highly sensitive [36]. This analysis enabled us to identify the parameters with the greatest impact on CER potential. On this basis, we systematically assessed the CER potential of PPP rooftop PV systems across a range of parameters.

### 2.1 Definitions

#### 2.1.1 Definition of functional zones within PPPs

We divided each plant into five functional zones to capture differences in production processes and carbon emissions. This zoning scheme adheres to Chinese national standards (i.e., the Code for the Design of Pulp and Papermaking Plants [37]) and was refined based on spatial patterns observed in high-resolution remote-sensing imagery. Areas not belonging to these five functional zones were classified as other built-up areas. The following list summarizes the functional roles, emission characteristics, and key visual features of PPP functional zones (see the Supplementary Fig. S1 for representative examples).

***Primary fiber stacking areas.*** These areas are used for the outdoor storage of raw primary fiber materials and serve as the upstream functional zone of the pulping process. They are associated with primary fiber pulping, which is characterized by high energy consumption and relatively high carbon emissions. These areas are typically rectangular or circular and are painted in yellow or dark brown.

***Recovered fiber stacking areas.*** These areas are used to store bundles of recovered fiber and appear as rectangular patterns. Recovered fiber pulping requires less energy and results in lower emissions compared with primary fiber pulping. Identification was based on spatial arrangement, spectral reflectance, and material color (typically yellow).

***Wastewater treatment areas****.* These areas contain sedimentation tanks, oxidation basins, and treatment pools. Water bodies vary in color depending on the treatment stage, with untreated wastewater appearing darker and treated effluent lighter. Emissions from this zone are mainly indirect. Structures are typically circular or square and form regular patterns.

***Thermal power plant areas***. These areas provide on-site energy supply and constitute the primary source of direct carbon emissions due to fuel combustion. They are characterized by tall chimneys and cooling towers with linear, circular, or elliptical structures, and they exhibit distinct spatial features in remote-sensing images.

***Other stacking areas***. These areas are used for outdoor storage of various materials and serve as a supporting role in the production system. Their emissions are relatively minor and mainly originate from material handling and short-distance transportation. In remote-sensing imagery, they appear as regular geometric patterns, most commonly rectangular, with diverse surface colors that distinguish them from surrounding areas.

***Other built-up areas****.* These areas include workshops, warehouses, offices, and related structures. Emissions from this zone are predominantly indirect and mainly result from electricity consumption. They are characterized by densely clustered buildings that form large, contiguous color blocks readily distinguishable from those of other functional zones.

#### 2.1.2 Definition of PPPs

We defined PPPs based on the delineation of functional zones combined with plant product information as follows:

***Primary fiber pulp plants (PFPPs).*** Plants characterized by the presence of primary fiber stacking areas on the plant sites (Supplementary Fig. S2a).

***Recovered fiber pulp plants (RFPPs).*** Plants characterized by the presence of recovered fiber stacking areas and the absence of primary fiber stacking areas (Supplementary Fig. S2b).

***Heavyweight paper product manufacturing plants (HPPMPs).*** Plants that lack both primary fiber and recovered fiber stacking areas, with production mainly focused on heavyweight paper products, excluding tissue and specialty papers (Supplementary Fig. S2c).

***Specialty paper product manufacturing plants (SPPMPs).*** Plants that lack both primary fiber and recovered fiber stacking areas and produce mainly specialty papers, such as wallpaper or filter paper (Supplementary Fig. S2d).

***Lightweight paper product manufacturing plants (LPPMPs).*** Plants that lack both primary fiber and recovered fiber stacking areas, with production mainly focused on lightweight paper products, primarily tissue paper (Supplementary Fig. S2e).

### 2.2 Data sources

In this research, we obtained the geographic coordinates of 720 PPPs located in China, which accounted for over 90% of the country’s paper production capacity in 2022, from the Baidu Maps Development Platform (https://lbsyun.baidu.com/). We collected remote-sensing images of the plants from Google Earth and sourced information on the main products they manufactured from the China Paper Industry Yearbook [38] and the official websites of the relevant companies. We extracted carbon emissions data from the plants' environmental, social, and governance (ESG) reports and solar resource data (specifically for annual total solar radiation) from the Resource and Environmental Science Data Platform (https://www.resdc.cn/Default.aspx) using a spatial resolution of 1 km. The detailed data for this study are available on Zenodo, an open-access repository for research data and code (https://zenodo.org/records/16629379).

### 2.3 Data preprocessing

**Fig. 2:**
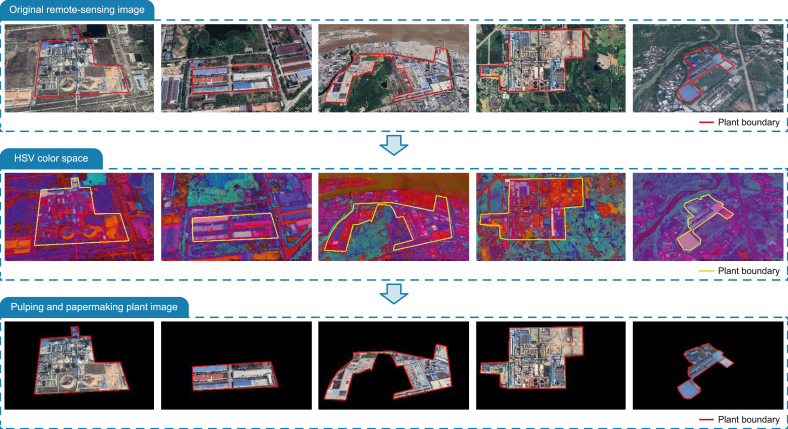
Examples of contour extraction for pulping and papermaking plants with different geometric shapes. Original remote-sensing images are converted to the hue–saturation–value (HSV) color space, followed by plant boundary extraction to produce images of pulping and papermaking plants.

To eliminate interference from surrounding buildings, we first delineated plant boundaries based on manual visual interpretation. We then converted the remote-sensing images from red–green–blue (RGB) color space to the hue–saturation–value (HSV) color space and thereafter performed contour extraction using OpenCV—an open-source computer vision library for image processing—to delineate PPP areas. To visually demonstrate the effectiveness of the contour extraction method, we selected several PPPs with different geometric shapes (Fig. 2). Finally, we annotated functional zones within each plant using LabelMe software, an open-source image annotation tool, following the zone definitions in Section 2.1.1.

To minimize potential interference from Chinese punctuation and rare characters on downstream classification, we preprocessed the textual data using the Jieba word-segmentation tool. Specifically, we normalized the original text by removing Chinese punctuation and infrequent characters to enhance the consistency of textual representations and the robustness of the classifier. We then classified and labeled PPPs by integrating information derived from remote-sensing images with plant-level product data.

### 2.4 Multimodal data fusion

#### 2.4.1 DeepLabv3+ model

**Fig. 3:**
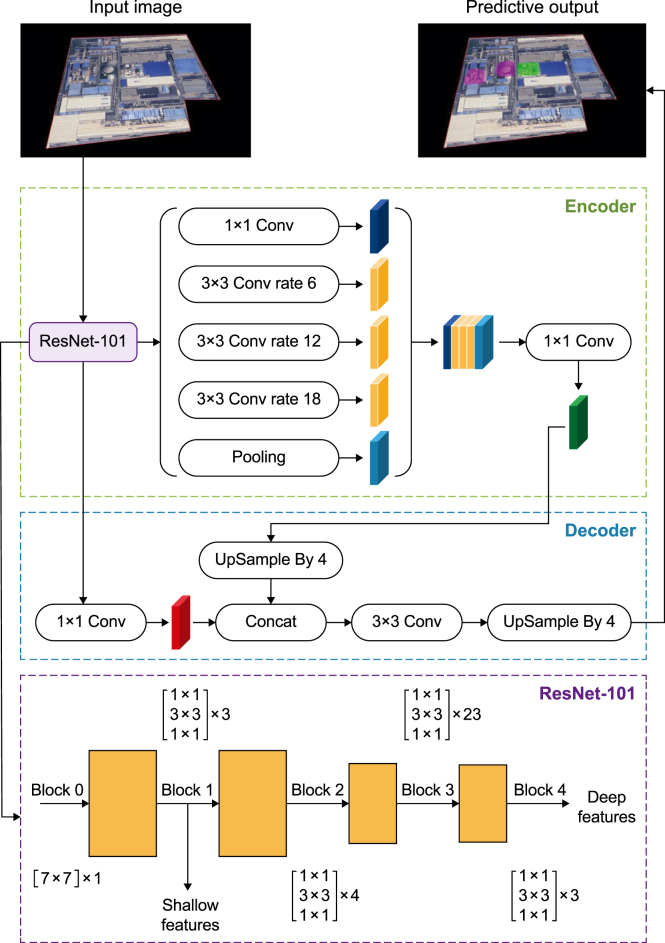
Architecture of the DeepLab**v3** + **model.** The DeepLabv3+ model adopts an encoder–decoder architecture. The encoder employs a ResNet-101 backbone integrated with atrous spatial pyramid pooling (ASPP) to capture multi-scale contextual information for semantic segmentation. The decoder fuses shallow and deep features to refine object boundaries and progressively up-samples feature maps to generate high-resolution segmentation maps. Conv: convolution.

The DeepLabv3+ model adopts an encoder–decoder architecture (Fig. 3) [39]. The encoder employs ResNet-101 as the backbone, comprising five blocks (Blocks 0–4) with 1, 3, 4, 23, and 3 residual blocks, respectively. For each input image, the backbone yields two feature streams: (i) shallow features from Block 1 (edge, texture, and color), which are fed directly into the decoder; and (ii) deep features from Block 4 that encode object categories and overall structure. The atrous spatial pyramid pooling (ASPP) module—four dilated convolution blocks and one pooling layer—further processed the deep features and generated five distinct feature outputs. These maps were then concatenated and passed through a 1 × 1 convolution layer before being fed into the decoder.

In the decoder, shallow features from the ResNet-101 encoder were first subjected to a 1 × 1 convolution layer. In parallel, the ASPP-derived deep semantic features were upsampled by a factor of four (linear interpolation) to restore the spatial resolution to the size of the shallow feature maps and ensure multiscale features. The transformed shallow features were concatenated with the upsampled deep features along the channel dimension and then refined by a 3 × 3 convolution layer. Finally, the decoder upsampled the resulting feature map was upsampled by an additional factor of four (bilinear interpolation) to recover the original input image resolution and produce a pixel-level semantic segmentation map.

#### 2.4.2 BERT model

**Fig. 4:**
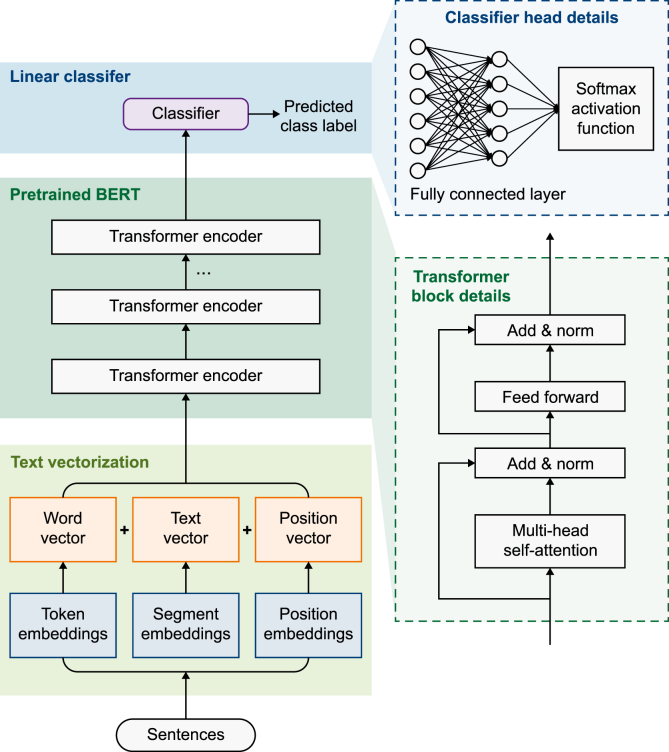
Architecture of the BERT model. Text inputs are vectorized using token, segment, and position embeddings and encoded by stacked Transformer layers. The final representations are fed into a softmax-linear classifier to generate predicted class labels. The two panels on the right show enlarged views of the Transformer block and the classifier head block. BERT: bidirectional encoder representations from the transformers.

The BERT architecture comprises three main components: a vector embedding layer, a pretrained BERT model, and a linear classifier [40]. The embedding layer transformed the textual input into word, text, and position vectors via token, segment, and position embeddings (Fig. 4). These representations were then fed into the pretrained BERT model. The pretrained BERT model consisted of multiple stacked transformer encoder layers, each with an identical architecture comprising two sublayers: a multihead attention mechanism and a feedforward neural network. The linear classifier included a fully connected layer and a Softmax activation function. We used the fully connected layer to map the output of the BERT model into raw score vectors (z*)* for different categories based on equation (1):

(1) $$\begin{array}{c} z=Wx+b \end{array}$$

where x is the output of the BERT model, W is the weight matrix, ***b*** is the bias vector, and z is the initial score vector.

Subsequently, we mapped z to the 0–1 range using the Softmax activation function to ensure that the sum of all elements equaled 1. Finally, we classified the text using equation (2):

(2) $$\begin{array}{c} p_{i}=\frac{\exp\left(z_{i}\right)}{\sum_{j=1}^{C}\exp\left(z_{j}\right)} \end{array}$$

where $p_{i}$ is the probability that the input sample belongs to class *i*, $z_{i}$ is the raw score of the input sample belonging to class *i*, and C is the number of classes in the classification (C = 5).

#### 2.4.3 Carbon accounting model

To better capture the relationship between functional area extents and the carbon emissions generated by PPPs, we developed an area-based carbon accounting model according to equation (3):

(3) $$\begin{array}{c} E_{c,i}=a_{i}\times A_{p,i}+b_{i}\times A_{r,i}+c_{i}\times A_{o,i}+d_{i}\times A_{w,i}+e_{i}\times A_{t,i}+f_{i}\times A_{b,i}+\varepsilon_{i} \end{array}$$

where $E_{c,i}$ refers to the carbon emissions of the *i*-th type of PPP, (ton of CO_2_); $A_{p,i}$, $A_{r,i}$, $A_{o,i}$, $A_{w,i}$, $A_{t,i}$, and $A_{b,i}$ represent the primary fiber stacking area, recovered fiber stacking area, other stacking areas, wastewater treatment area, thermal power plant area and other built-up areas of the *i-th* type of PPP, respectively (m^2^); the coefficients $a_{i}$, $b_{i}$, $c_{i}$, $d_{i}$, $e_{i}$, and $f_{i}$ represent the respective contributions of the five functional zones to the overall carbon emissions; $\varepsilon_{i}$ is the error term.

### 2.5 Model evaluation metrics

To evaluate the classification model's performance, we used accuracy, precision, recall, and the F1 score as evaluation metrics, which are denoted by U, P, R, and $F_{1}$, respectively. Their formulations are given in equations (4), (5), (6), (7):

(4) $$\begin{array}{c} U=\frac{N_{\text{TP}}+N_{\text{NP}}}{N_{\text{TP}}+N_{\text{TN}}+N_{\text{FP}}+N_{\text{FN}}}\times 100\% \end{array}$$

(5) $$\begin{array}{c} P=\frac{N_{\text{TP}}}{N_{\text{TP}}+N_{\text{FP}}}\times 100\% \end{array}$$

(6) $$\begin{array}{c} R=\frac{N_{\text{TP}}}{N_{\text{TP}}+N_{\text{FP}}}\times 100\% \end{array}$$

(7) $$\begin{array}{c} F_{1}=\frac{2\times P\times R}{P+R} \end{array}$$

where $N_{\text{TP}}$ (true positives) are true examples, $N_{\text{TN}}$ (true negatives) are true counterexamples, $N_{\text{FP}}$ (false positives) are false positive examples, and $N_{\text{FN}}$ (false negatives) are false negative examples.

For the semantic segmentation model based on remote-sensing images, we employed the intersection over union (*IoU*) to measure the similarity between the model’s segmentation results and the true labels (equation (8)).

(8) $$\begin{array}{c} {IoU}_{k}=\frac{S_{k}\cap G_{k}}{S_{k}\cup G_{k}} \end{array}$$

In this equation, *IoU*_*k*_ denotes the intersection over union for class *k*, $S_{k}$ is the pixel area predicted by the model, and $G_{k}$ is the annotated pixel area.

For the carbon emission regression model, we employed the coefficient of determination (*R*^*2*^, equation (9)) and the mean absolute percentage error (*MAPE*, equation (10)) as evaluation metrics.

(9) $$\begin{array}{c} R^{2}=1-\frac{\sum_{i=1}^{n}{\left(y_{i}-{\hat{y}}_{i}\right)}^{2}}{\sum_{i=1}^{n}{\left(y_{i}-\bar{y}\right)}^{2}} \end{array}$$

(10) $$\begin{array}{c} MAPE=\frac{1}{n}\sum_{i=1}^{n}\left|\frac{y_{i}-{\hat{y}}_{i}}{y_{i}}\right|\times 100\% \end{array}$$

In these equations, $y_{i}$, ${\hat{y}}_{i}$, and $\bar{y}$ refer to the actual, predicted, and average carbon emissions of the *i*-th plant, respectively, and *n* is the total number of samples.

### 2.6 Assessment of CER potential

To assess the CER potential of rooftop PV power generation for existing PPPs, we assumed that all other built-up areas were available for PV panel installation. For the modeling calculations, we followed the approach described by Wang et al. [41] (equations (11), (12)):

(11) $$\begin{array}{c} \beta_{\text{opt}}={-0.0049\times \varphi }^{2}+1.088\times \varphi \end{array}$$

(12) $$\begin{array}{c} d_{s}=L_{\text{pv}}\times \cos\;\beta_{\text{opt}}+\frac{\left(L_{\text{pv}}\times \sin\;\beta_{\text{opt}}\right)}{\tan\left(66.55^{\circ }-\varphi \right)} \end{array}$$

where $\beta_{\text{opt}}$ is the optimal radiation angle (°), $d_{s}$ is the spacing between PV panels (m), φ is the latitude of the plant location (°), and $L_{\text{pv}}$ is the length of the PV panel (m).

We then calculated the roof-mounted PV power generation $E_{\text{pv}}$ (kWh) using equations (13), (14):

(13) $$\begin{array}{c} A_{\text{pv}}=A_{a}\times \frac{1}{d_{s}} \end{array}$$

(14) $$\begin{array}{c} E_{\text{pv}}=\theta \times A_{\text{pv}}\times H_{T}\times \delta \times \frac{1-F_{s}}{3.6} \end{array}$$

where $A_{\text{pv}}$ represents the area of the rooftop PV panels (m^2^), $A_{a}$ is the total area of the rooftop (m^2^), $H_{T}$ is the total solar radiation intensity at the plant location (MJ m^−2^ year^−1^), θ is the efficiency of PV modules, and $F_{s}$ is the shading coefficient. δ is the performance ratio (set to 0.8 following Wang et al. [41]). We set θ = 15% and $F_{s}$ = 0.05, following Wang et al. [42]. MJ was converted to kWh by dividing by 3.6.

Finally, to calculate CER ($C_{\text{pv}}$), we used the following equation:

(15) $$\begin{array}{c} C_{\text{pv}}=\left(Q_{\text{coal}}-Q_{\text{pv}}\right)\times E_{\text{pv}} \end{array}$$

where $Q_{\text{coal}}$ and $Q_{\text{pv}}$ are the life cycle for coal-fired and PV power generation (g CO_2_ kWh^−1^), respectively. According to IPCC [43], we used $Q_{\text{coal}}$ = 950 g CO_2_ kWh^−1^ and $Q_{\text{pv}}$ = 50 g CO_2_ kWh^−1^.

### 2.7 GSA method

GSA was conducted using a variance-based approach that allows for correlated input parameters. The model output is expressed as:

(16) $$\begin{array}{c} Y=g\left(X_{1},X_{2},\ldots ,X_{n}\right) \end{array}$$

where Y is the model output and $X_{1},X_{2},\ldots ,X_{n}$ represent the uncertain input parameters.

Assuming the model is executed *M* times, the sample variance is calculated as follows:

(17) $$\begin{array}{c} \text{Var}\left(Y\right)=\frac{1}{M}\sum_{j=1}^{M}{\left(Y_{j}-\bar{Y}\right)}^{2} \end{array}$$

where $Y_{j}$ is the result of the *j*-th model run, and $\bar{Y}$ represents the mean value obtained from *M* model runs.

Finally, we used equations (18), (19) to calculate the first-order and total-order Kucherenko sensitivity indices [44]:

(18) $$\begin{array}{c} S_{i}=\frac{\text{Var}\left(\bar{Y}|X_{i}\right)}{\text{Var}\left(Y\right)} \end{array}$$

(19) $$\begin{array}{c} S_{T,i}=1-\frac{\text{Var}\left(\bar{Y}|X_{∼i}\right)}{\text{Var}\left(Y\right)} \end{array}$$

where $S_{i}$ is the first-order Kucherenko sensitivity index of input paramete, which quantifies the contribution of $X_{i}$ to the total output variance while allowing for correlated inputs. The total-effect Kucherenko sensitivity index $S_{T,i}$ denotes the total-effect Kucherenko sensitivity index reflects the overall influence of $X_{i}$ on output uncertainty, including all interaction and correlation effects. $\bar{Y}|X_{i}$ represents the mean model output obtained when the input parameter $X_{i}$ is fixed at a given value. $\bar{Y}|X_{∼i}$ is the mean model output obtained when all input parameters except $X_{i}$ fixed, $X_{∼i}$ represents the set of all input parameters excluding $X_{i}$.

## 3 Results and discussion

### 3.1 Classification results

#### 3.1.1 Results of the DeepLabv3+ model

**Fig. 5:**
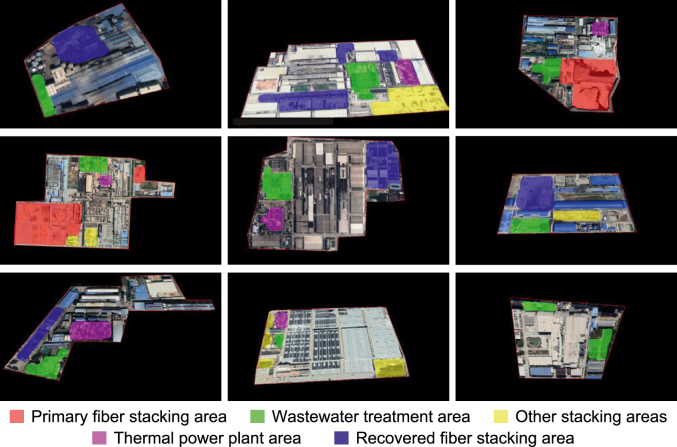
Semantic segmentation results for pulping and papermaking plants. Different functional zones are identified: primary fiber stacking (red), wastewater treatment (green), recovered fiber stacking (blue), other stacking (yellow), and thermal power plant areas (purple).

We used 720 preprocessed remote-sensing images of PPPs for this study, allocating 70% to the training set and 30% to the test set. We applied five functional zones (defined in Section 2.1.1) and visualized them using distinct colors (Fig. 5): primary fiber stacking (red), wastewater treatment (green), recovered fiber stacking (blue), other stacking (yellow), and thermal power plant areas (purple).

The model achieved high classification performance, with recognition accuracies exceeding 90% across all five functional-zones categories (Table 1). The highest recognition accuracy (98.58%) was achieved in the primary fiber-stacking areas, reflecting the presence of highly distinctive visual features. In addition, *IoU* values for all functional zones exceeded 80%, indicating that the model effectively captured boundary characteristics and discriminated among different functional zones.

**Table 1:** Accuracy metrics of semantic segmentation models applied to factory functional zones.

Functional zone	*U*	*P*	*R*	*F*_1_	*IoU*
Primary fiber stacking area	98.58%	93.84%	93.93%	0.93	94.00%
Recovered fiber stacking area	96.45%	93.84%	85.29%	0.89	83.53%
Wastewater treatment area	95.39%	92.31%	94.96%	0.93	86.39%
Other stacking areas	92.55%	88.23%	77.65%	0.82	81.85%
Thermal power plant area	92.90%	90.77%	83.19%	0.86	86.32%

Note: *U*, *P*, *R*, *F*_1_, and *IoU* are accuracy, precision, recall, *F*_1_-score, and intersection over union, respectively. An *F*_1_ score closer to 1 denotes better model performance. PFPP, primary fiber pulp plant; RFPP, recovered fiber pulp plant; HPPMP, heavyweight paper product manufacturing plant; SPPMP, specialty paper product manufacturing plant; LPPMP, lightweight paper product manufacturing plant.

Based on this model, we identified the functional zones of 720 PPPs from remote-sensing images. Among these plants, 92 were classified as PFPPs and 108 as RFPPs.

#### 3.1.2 Results of BERT model

The model exhibited strong performance across the three categories (HPPMP, LPPMP, and SPPMP), with classification accuracies of 91.62%, 84.55%, and 85.62%, respectively (Table 2). Precision and recall values for all three categories exceeded 80%. These results confirmed that the model effectively captured the distinctive textual features relating to different plant types.

**Table 2:** Evaluation metrics for text classification models.

Plant type	*U*	*P*	*R*	*F*_1_
PFPP	66.38%	80.26%	66.30%	0.73
RFPP	73.82%	62.77%	81.90%	0.71
HPPMP	91.62%	88.27%	88.81%	0.88
LPPMP	84.55%	84.39%	85.28%	0.84
SPPMP	85.62%	86.26%	85.60%	0.86

Note: *U*, *P*, *R*, and *F*_1_, are accuracy, precision, recall, and *F*_1_-score, respectively. An *F*_1_ score closer to 1 denotes better model performance. PFPP, primary fiber pulp plant; RFPP, recovered fiber pulp plant; HPPMP, heavyweight paper product manufacturing plant; SPPMP, specialty paper product manufacturing plant; LPPMP, lightweight paper product manufacturing plant.

The incorporation of textual information substantially improved the classification model's accuracy. The classification of HPPMPs, LPPMPs, and SPPMPs based solely on remote-sensing images is challenging, owing to the limited discriminative visual features of such images. Hence, the textual data provided critical complementary information to overcome these limitations. The multimodal fusion of image and textual data effectively overcame the constraints inherent in single-modality PPP classification.

Using a classification framework that integrated semantic image segmentation with textual information, we categorized the 720 PPPs by type. HPPMPs constituted the largest group (292 plants; >40% of the total), followed by SPPMPs (122), RFPPs (108), and LPPMPs (106), whereas PFPPs represented the smallest category (92).

### 3.2 Carbon accounting model

Due to potential variations in data accuracy and reporting standards in the reported ESG data, we examined the carbon emissions data prior to model construction and conducted field surveys at a set of representative PPPs to validate the reported emissions values. The survey-based estimates were generally consistent with the reported ESG data (Table 3), with discrepancies predominantly within 10%. Therefore, despite potential uncertainties, we considered the ESG data reliable enough for use in this study.

**Table 3:** Comparison between ESG-reported and surveyed carbon emissions for representative PPPs.

Plant type	Surveyed mean values (t CO_2_ per t paper)	ESG-reported mean values (t CO_2_ per t paper)
PFPP	1.09	1.03
RFPP	0.71	0.78
HPPMP	0.52	0.58
LPPMP	0.93	0.92
SPPMP	0.81	0.8

Note: PFPP, primary fiber pulp plant; RFPP, recovered fiber pulp plant; HPPMP, heavyweight paper product manufacturing plant; SPPMP, specialty paper product manufacturing plant; LPPMP, lightweight paper product manufacturing plant; ESG, environmental, social, and governance.

Based on the carbon emissions data collected from PPPs, combined with the classification results from Section 3.1, we used the methods described in Section 2.6 to construct area-based carbon accounting models for five different PPP types defined above. Overall, the models exhibited satisfactory fitting performance, with all *R*^*2*^ values exceeding 0.75 (Table 4). Of the five models, the RFPP model achieved the highest fitting accuracy (*R*^*2*^ = 0.96, *MAPE* = 8.10%). In contrast, the SPPMP and HPPMP models generated relatively higher *MAPE* values, both exceeding 19%. This increase was mainly attributable to the number of individual plants with exceptionally high carbon emissions within these categories (Supplementary Fig. S3), which led to elevated prediction errors and, consequently, higher overall *MAPE* values. However, because some enterprises with similar high-emission plants lack publicly disclosed ESG reports, we did not exclude these outliers to maintain the model's representativeness. Overall, the predicted emissions agreed well with the observed values for most of the samples. Thus, we subsequently applied the confirmed models to conduct plant-level carbon accounting for 720 classified PPPs. In future work, we plan to further improve model performance by using more balanced training samples and advanced feature engineering to better capture this variability.

**Table 4:** Carbon emission accounting model evaluation indicators.

Plant type	*R*^2^	*MAPE*
PFPP	0.90	9.95%
RFPP	0.96	8.10%
LPPMP	0.88	12.68%
SPPMP	0.75	19.21%
HPPMP	0.76	19.49%

Note: *R*^2^, coefficient of determination with values closer to 1 indicates better model performance; *MAPE*, mean absolute percentage error; PFPP, primary fiber pulp plant; RFPP, recovered fiber pulp plant; HPPMP, heavyweight paper product manufacturing plant; SPPMP, specialty paper product manufacturing plant; LPPMP, lightweight paper product manufacturing plant.

Using the confirmed models, we analyzed the contributions of different functional zones to plant-level carbon emissions (Table 5). Across all PPP categories, wastewater treatment areas consistently contributed to higher carbon emissions. This pattern showed that increases in production capacity were accompanied by substantial growth in wastewater generation, necessitating larger wastewater treatment systems and, consequently, generating higher carbon emissions. This relationship was particularly pronounced for PFPPs, reflecting their high water consumption and pollution intensity during pulping, which, in turn, led to greater reliance on wastewater treatment infrastructure.

**Table 5:** Driving effects of different functional area sizes on plant carbon emissions.

Plant type	*a*	*b*	*c*	*d*	*e*	*f*	ε
PFPP	−0.54	0.47	9.70	14.93	−0.36	−0.58	50.40
RFPP	-	0.24	0.00	1.86	0.00	1.75	−13.93
LPPMP	-	-	0.20	5.53	0.00	0.61	7.91
SPPMP	-	-	−3.76	7.61	14.740	0.83	−0.41
HPPMP	-	-	0.24	1.86	0.00	1.74	−1.74

Note: *a*, *b*, *c*, *d*, *e*, and *f* denote the regression coefficients corresponding to the primary fiber stacking area, recovered fiber stacking area, other stacking areas, wastewater treatment area, thermal power plant area, and other built-up areas, respectively, indicating their contributions to overall carbon emissions. ***ε*** denotes the error term. PFPP, primary fiber pulp plant; RFPP, recovered fiber pulp plant; HPPMP, heavyweight paper product manufacturing plant; SPPMP, specialty paper product manufacturing plant; LPPMP, lightweight paper product manufacturing plant.

Beyond wastewater treatment areas, the influence of different functional zones on carbon emissions varied markedly across plant types. The thermal power plant areas, for instance, were positively associated with carbon emissions only for some plant categories, with the effect being particularly pronounced for SPPMPs. This pattern could be attributed to the relatively small scale of such plants, in which thermal power plants often occupy a large proportion of the total site area and therefore exert a stronger influence on overall carbon emissions. In contrast, for larger plants, such as PFPPs and RFPPs, although thermal power plants remained an important source of direct carbon emissions, their spatial footprints typically accounted for smaller proportions of the total site areas. Consequently, their contributions to carbon emissions were small within the area-based model. Overall, these results highlight pronounced heterogeneity across functional zones shaping plant-level carbon emissions across PPP categories.

### 3.3 PPP carbon emission inventory

**Fig. 6:**
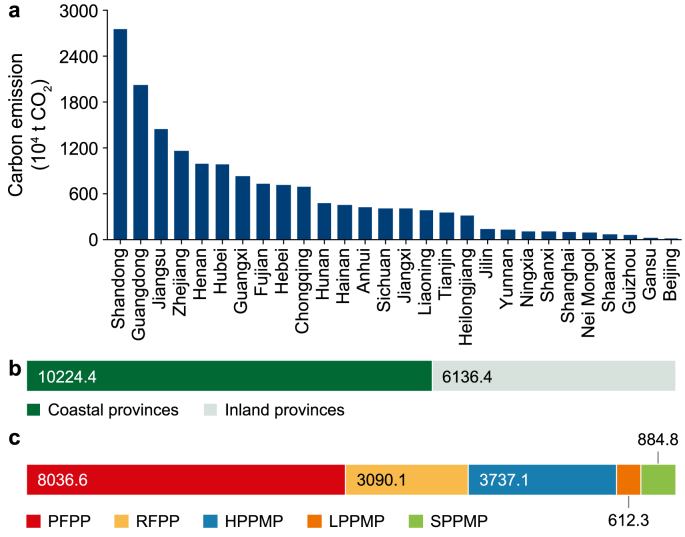
Distribution of carbon emissions from pulping and papermaking plants in China **(2022). a,** Provincial distribution of carbon emissions. **b,** Comparison of total carbon emissions between coastal and inland provinces. **c,** Total carbon emissions of different plant types, showing the contribution of each plant category to overall emissions. PFPP: primary fiber pulp plant, RFPP: recovered fiber pulp plant, HPPMP: heavyweight paper product manufacturing plant, SPPMP: specialty paper product manufacturing plant, LPPMP: lightweight paper product manufacturing plant.

In 2022, total carbon emissions from China’s PPI were approximately 163.6 million tons of CO_2_, with a heterogeneous spatial distribution across 720 PPPs (Supplementary Fig. S4). Overall, 720 PPPs were distributed across 28 Chinese provinces and municipalities, with pronounced regional disparities in carbon emissions generated by the PPI (Fig. 6a). From a regional perspective, China’s PPPs are mainly concentrated in coastal provinces. The 10 coastal regions (Guangdong, Shandong, Jiangsu, Zhejiang, Hainan, Fujian, Shanghai, Guangxi, Liaoning, and Tianjin) collectively contributed 102.3 million tons of CO_2_ to PPI carbon emissions, accounting for 62.3% of the national total (Fig. 6b). Only Guangdong and Shandong reported carbon emissions exceeding 20 million tons of CO_2_ (Fig. 6a). Combined, they generated more than one-quarter of China’s total PPI carbon emissions. In contrast, most inland provinces reported emissions below 5 million tons of CO_2_.

**Fig. 7:**
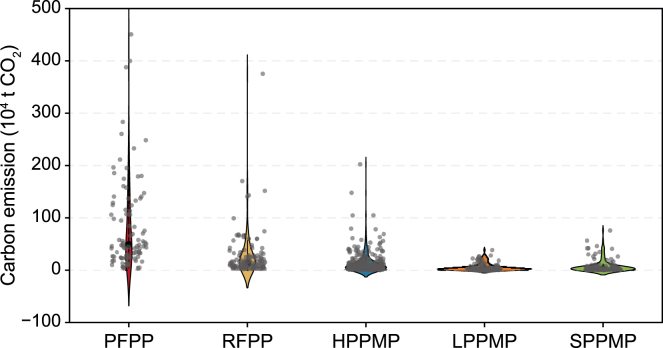
Carbon emissions from pulping and papermaking plants producing different products. Violin plots represent the distribution of carbon emissions for each plant, scattered points indicate individual data points, and black circles indicate the median values. For primary fiber pulp plant (PFPP), recovered fiber pulp plant (RFPP), heavyweight paper product manufacturing plant (HPPMP), lightweight paper product manufacturing plant (LPPMP), and specialty paper product manufacturing plant (SPPMP), the minimum–maximum carbon emissions were (4.36–450.70) × 10^4^, (2.33–375.34) × 10^4^, (0.34–202.38) × 10^4^, (0.32–38.48) × 10^4^, and (0.30–75.84) × 10^4^ t CO_2_, respectively.

Total emissions from PFPPs, RFPPs, and HPPMPs amounted to 80.4, 30.9, and 37.4 million tons of CO_2_, respectively, accounting for 90.4% of national PPI emissions (Fig. 6c). Carbon emissions from PFPPs and RFPPs displayed strongly right-skewed distributions (Fig. 7), with maximum values far exceeding those for the other categories. This indicates the presence of extremely high-emission individual plants, which significantly elevates the overall carbon emission levels of the sector. In contrast, carbon emissions from LPPMPs and SPPMPs exhibited more concentrated distributions, with lower mean values and reduced extremes. This suggests relatively smaller disparities in carbon emissions among enterprises within these plant categories.

There was also considerable variation in carbon emissions among plants that produced similar products. Taking PFPPs as an example, individual plant emissions ranged from 43,600 to 450,700 tons of CO_2_, representing a difference of more than two orders of magnitude. This pronounced skewness indicates that a small number of large, high-emission plants contribute disproportionately to total sectoral emissions, resulting in a strongly nonlinear emissions structure. For instance, the Jinhai Pulping and Papermaking Plant in Hainan emitted 450,700 tons of CO_2_—approximately five times the PFPP category average (87,350 tons of CO_2_)—and accounted for 5.6% of total PFPP emissions.

Further statistical analysis revealed that approximately 5% of high-emission PPPs accounted for approximately 43% of total PPI carbon emissions. In contrast, nearly 75% of PPPs exhibited carbon emissions below the sectoral average, suggesting that most operated with relatively low emission intensities. These findings underscore the importance of adopting a differentiated approach to CER. While maintaining sector-wide coordination, regulatory efforts, and technical upgrades should be prioritized for a small subset of high-emission plants to support more efficient and cost-effective decarbonization.

## 4 Results for CER potential in the PPI

China is a vast country with complex terrain, resulting in significant variations in solar energy resources across regions (Supplementary Fig. S5). In general, solar radiation levels are higher in western regions than in central and eastern regions. Therefore, assessments of the potential of solar power generation for PPPs must explicitly account for regional variations in solar resource availability across plant locations. The annual total solar radiation at the locations of PPPs consistently exceeded 4000 MJ m^−2^ year^−1^ (Supplementary Fig. S6), with most values concentrated between 5000 and 6000 MJ m^−2^ year^−1^. This distribution indicates substantial potential for PV power generation across most PPP locations.

According to the calculation framework described in Section 2.6 and the GSA of potential CER due to PV power generation (Supplementary Fig. S7), only the first-order and total sensitivity indices for PV panel length exceeded the 0.1 threshold. Both were substantially higher than those for all other variables. This indicates that panel length is the dominant factor influencing CER outcomes. Accordingly, we further evaluated the rooftop PV power-generation potential of PPPs across four scenarios representing different installation densities. In these scenarios, all model parameters were held constant, whereas the PV panel lengths (*L*_p_ᵥ) were set to 0.5, 1.0, 1.5, and 2.0 m, respectively.

**Fig. 8:**
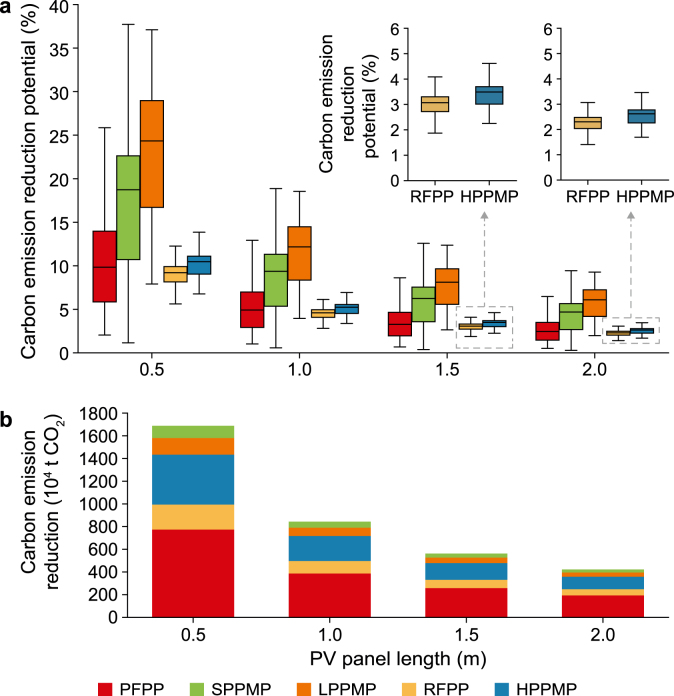
Carbon emission reduction potential under different photovoltaic panel lengths. a, Box plots show the distribution of carbon-emission-reduction potential for different plant types at varying photovoltaic (PV) panel lengths. The boxes represent the interquartile range, the central line indicates the median value, and the error bars denote the minimum and maximum values. The inset figures show magnified views of the recovered fiber pulp plant (RFPP) and heavyweight paper product manufacturing plant (HPPMP) at PV panel lengths of 1.5 m and 2.0 m, to better illustrate their differences in the low-value range. **b,** Total carbon emission reduction of different plant types under different PV panel lengths. PFPP: primary fiber pulp plant, SPPMP: specialty paper product manufacturing plant, LPPMP: lightweight paper product manufacturing plant.

As the panel length increased, the CER potential consistently declined across all plant types (Fig. 8a). The greatest carbon-reduction benefits were observed when the panel length was set to 0.5 m, with some facilities achieving potential reductions exceeding 35%. According to this optimal scenario, LPPMPs and SPPMPs had particularly high CER potential at the individual level, with most plants exceeding 20%. The CER potential of individual RFPP and HPPMP plants was relatively low across all scenarios, generally below 10%, and in some plants, the potential approached 0% for 1.5 and 2.0 m panel lengths.

We further quantified the overall CER potential of China’s PPI (Fig. 8b). Across the four scenarios, projected emissions reductions amounted to 16.9, 8.4, 5.6, and 4.2 million tons of CO_2_, corresponding to 10.3%, 5.2%, 3.4%, and 2.6% of total PPI emissions, respectively. Across plant types, PFPPs exhibited the highest CER potential in all scenarios, accounting for more than 40% of the total reduction. These results indicate that prioritizing the deployment of PV systems across PFPPs could deliver the largest sectoral-level carbon mitigation benefits.

In addition, although individual HPPMPs exhibited relatively low CER potential, their large number and extensive aggregate land areas mean that their cumulative CER potential cannot be ignored. In contrast, SPPMPs and LPPMPs tended to show relatively high CER potential at the individual plant level. These plants typically operate at lower emission levels and occupy smaller site areas, resulting in comparatively limited cumulative CER potential at the sectoral scale.

## 5 Conclusion

Under China’s “dual carbon” strategy, which targets carbon emission peaking by 2030 and carbon neutrality by 2060, carbon emissions management in key industries has been increasingly strengthened. However, researchers have primarily relied on provincial- or sector-level data, paying limited attention to plant-level analyses and mitigation pathways. To address this gap, we developed a multimodal data fusion framework for PPPs that integrates remote-sensing imagery, textual, and numerical data to improve plant-level carbon accounting and identify the key functional zones that generate emissions. In addition, we systematically evaluated the CER potential of rooftop PV systems to provide quantitative support for the low-carbon transition in China’s PPI. The main conclusions of this study are summarized as follows:

(1) In this study, we developed a multimodal data fusion framework for China’s PPI by integrating remote-sensing imagery and plant textual data. Model validation across five plant types demonstrated robust performance, with *R*^2^ values ranging from 0.75 to 0.96 and *MAPE* values ranging from 8.10% to 19.49%.
(2) Based on multimodal data fusion framework, 720 PPPs across China generated an estimated 163.6 million tons of CO_2_ in 2022, with more than 60% emitted from nine eastern coastal provinces. Significant differences were also observed across plant types and individual plants. PFPPs, RFPPs, and HPPMPs accounted for 90.2% of total carbon emissions, with the top 10% of high-emission plants contributing nearly half of sectoral carbon emissions. These findings provide a quantitative basis for differentiated region-specific CER strategies.
(3) Building on the emissions assessment, we developed a methodological framework to evaluate rooftop PV potential for PPPs using meteorological data and GSA. The scenario results revealed that a PV panel length of 0.5 m yielded the greatest emissions mitigation, with an annual reduction of approximately 16.9 million tons of CO_2_, corresponding to 10.3% of total sectoral emissions. This highlights rooftop PV deployment as a promising and scalable decarbonization strategy for the PPI.

## CRediT authorship contribution statement

**Song Hu:** Writing – original draft, Methodology, Formal analysis. **Huaqing Qi:** Data curation, Software. **Zifei Wang:** Data curation, Validation. **Xiaoyu Wu:** Data curation. **Yulin Han:** Writing – review & editing, Funding acquisition. **Yi Man:** Supervision, Conceptualization, Funding acquisition, Writing – review & editing.

## Declaration of competing interest

The authors declare that they have no known competing financial interests or personal relationships that could have appeared to influence the work reported in this paper.
