# Supplementary material for: Plant-level carbon accounting of China's pulp and paper industry via multimodal fusion
Source: Environ Sci Ecotechnol. 2026 Mar 6;30:100682. doi: 10.1016/j.ese.2026.100682 (PMC12997205; doi:10.1016/j.ese.2026.100682)
Supplement: Multimedia component 1 [file mmc1.docx]

**Plant-level carbon accounting of China's pulp and paper industry via multimodal fusion**

Song Hu^a^, Huaqing Qi^a^, Zifei Wang^a^, Xiaoyu Wu^b^, Yulin Han^a,^*, Yi Man^a,^*

^a^ State Key Laboratory of Advanced Papermaking and Paper-Based Materials, South China University of Technology, Guangzhou, 510640, China

^b^ School of Chemistry and Chemical Engineering, South China University of Technology, Guangzhou, 510640, China

*Corresponding Author:

manyi@scut.edu.cn (Yi Man)

[linyv@scut.edu.cn (Yulin](mailto:linyv@scut.edu.cn%20(Yulin) Han)

Summary Information

Number of pages: 4

Figures: S1-S7


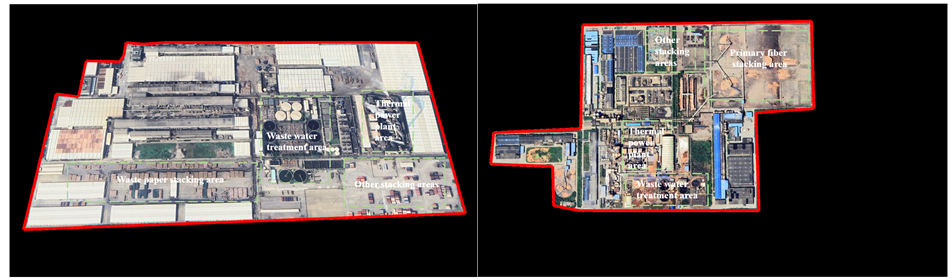


**Fig. S1. Schematic Diagram of Functional Zoning in Typical PPPs**


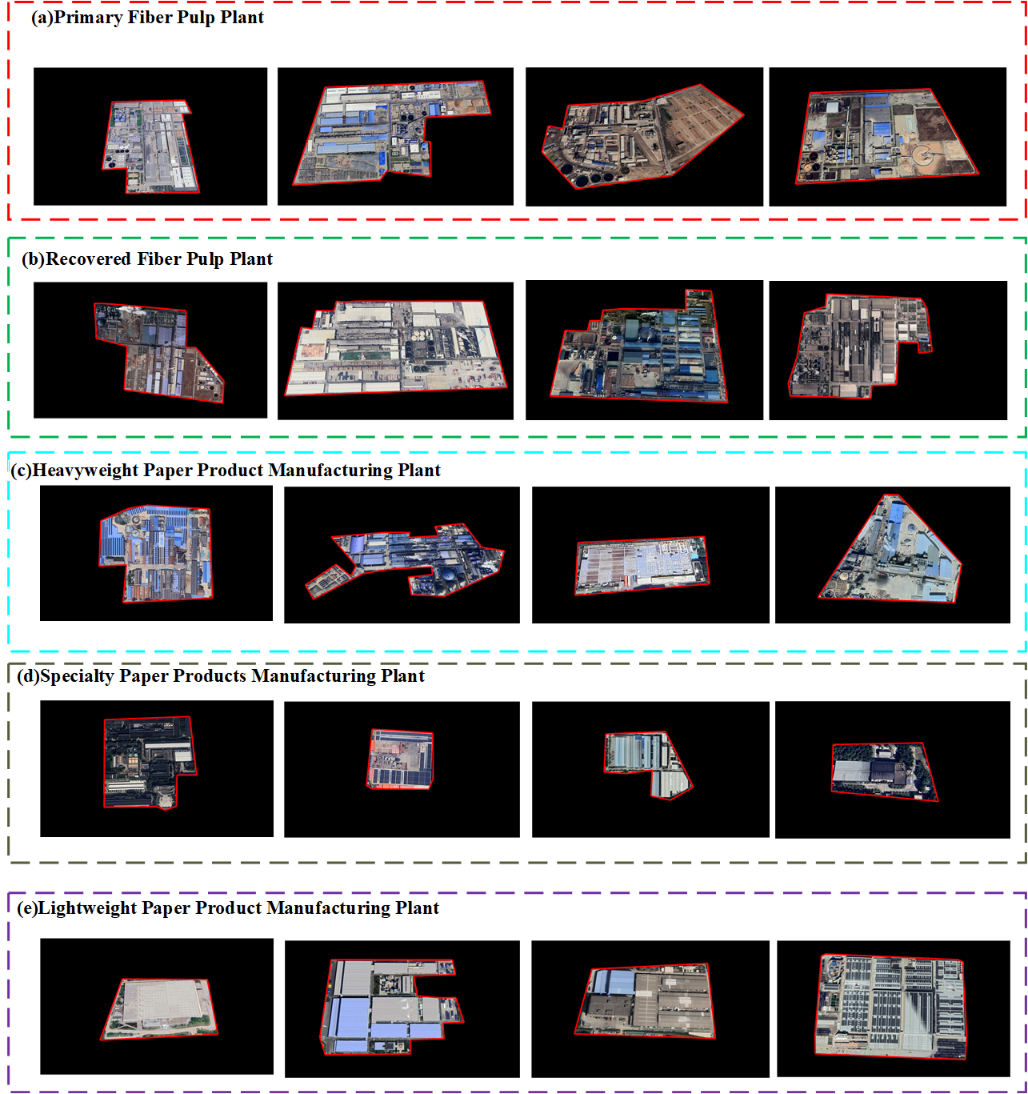


**Fig. S2. Representative Remote Sensing Images of Five Types of PPPs**


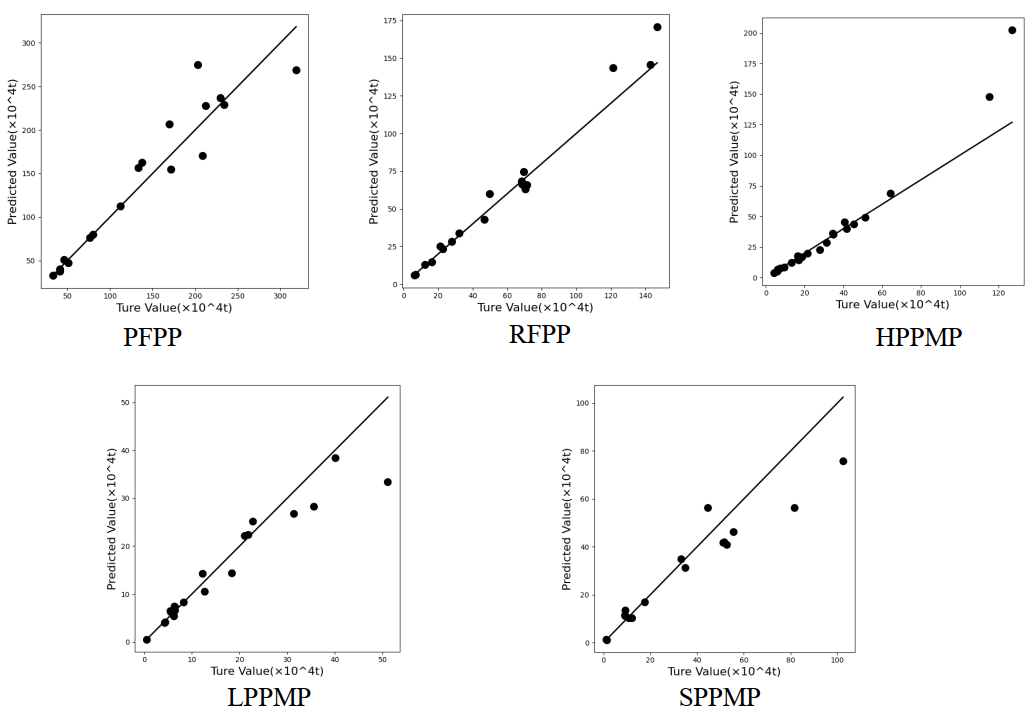


**Fig. S3. Comparison of fitted values and actual values in carbon emission calculation models**


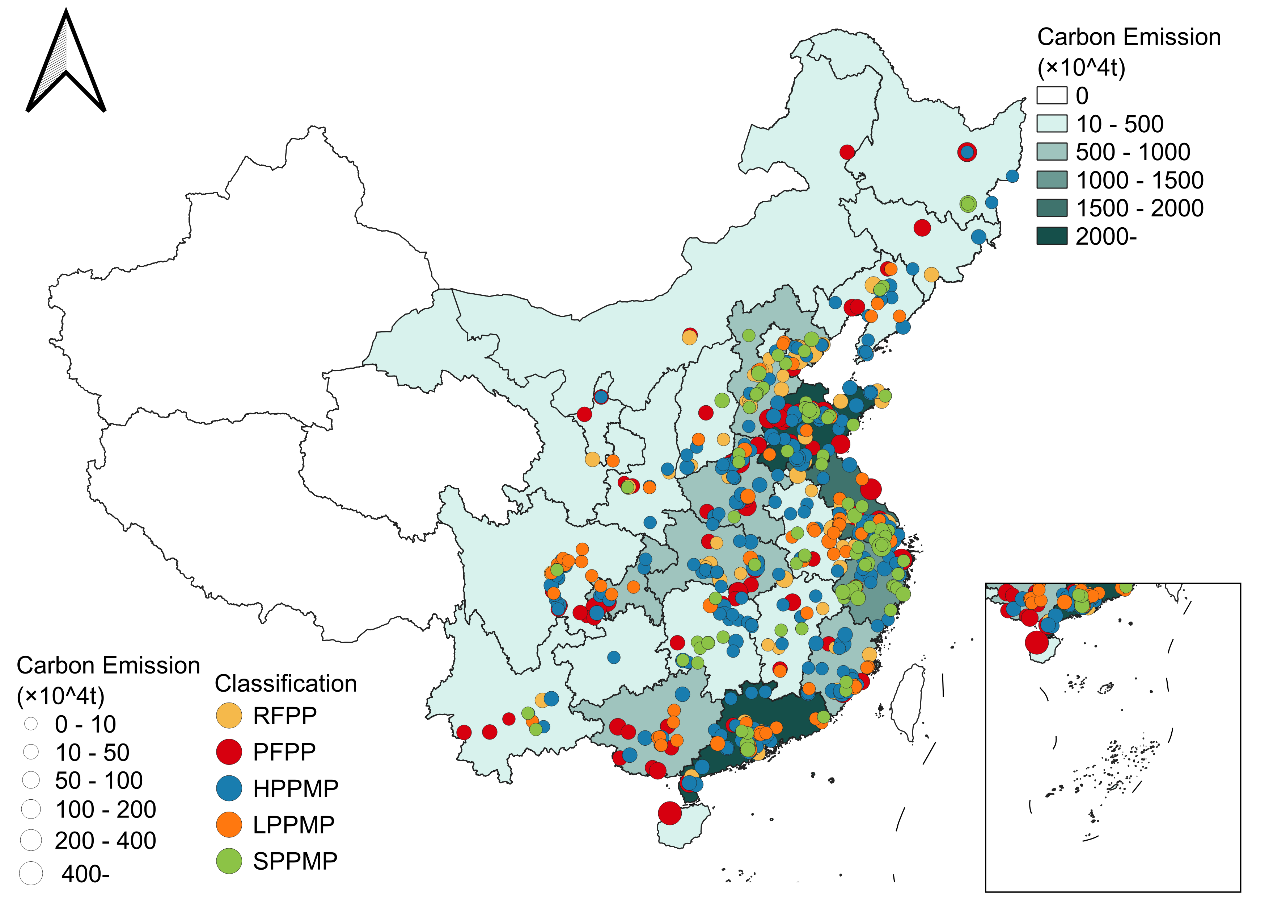


**Fig. S4. Spatial distribution of plant-level carbon emissions**


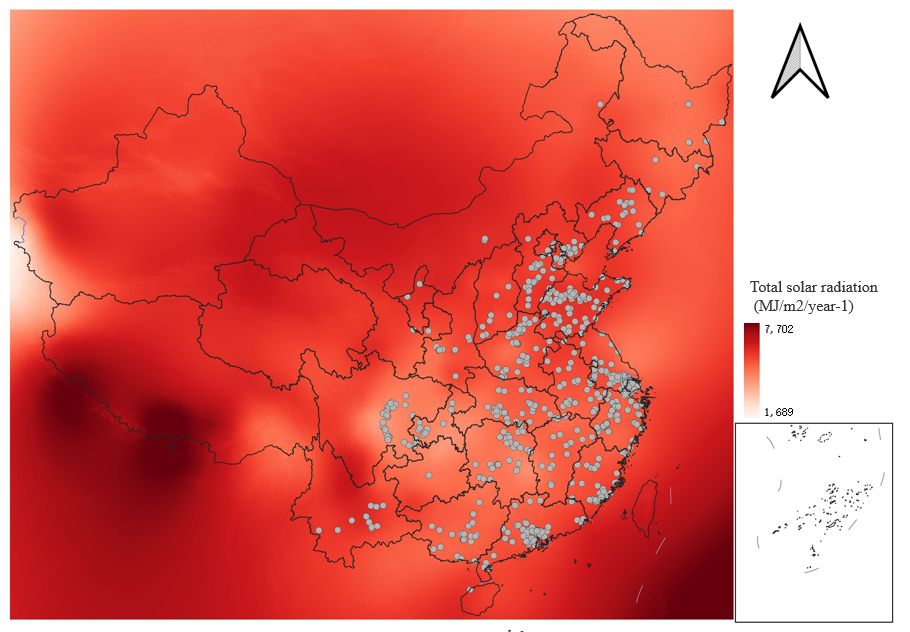


**Fig. S5**. **Distribution of solar energy resources and PPPs in China**


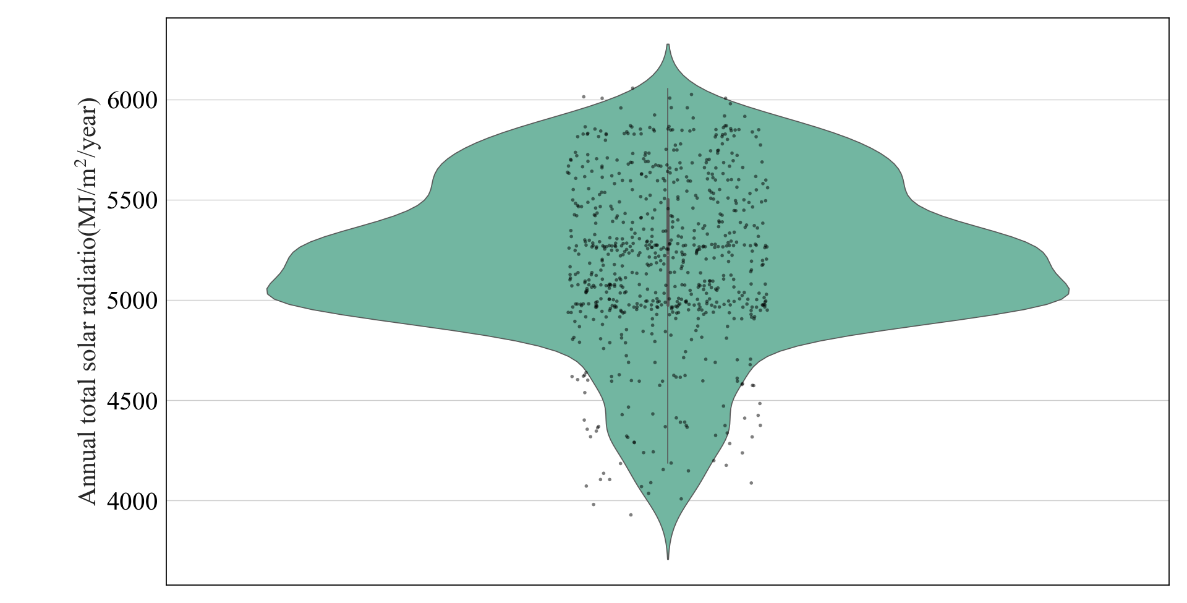


**Fig. S6. Solar energy resources in the area where the PPPs is located**


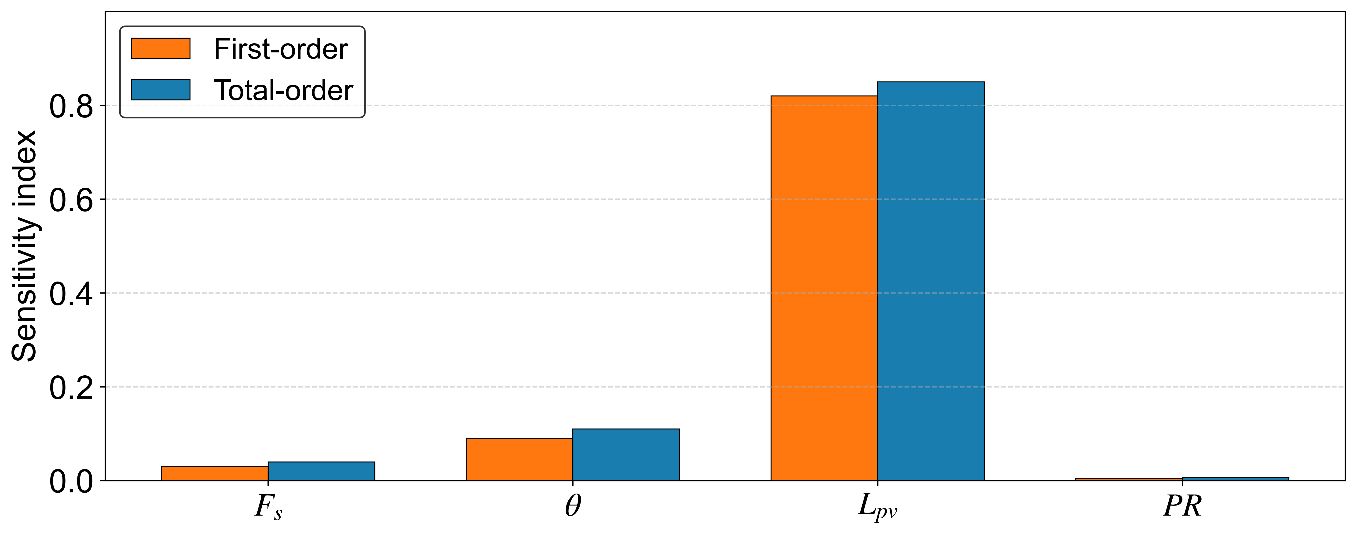


**Fig. S7. Sensitivity analysis results**
